# iRhom2 deficiency reduces sepsis-induced mortality associated with the attenuation of lung macrophages in mice

**DOI:** 10.1007/s00418-024-02318-5

**Published:** 2024-08-12

**Authors:** Jihye Kim, Jee Hyun Kim, Younghoon Kim, Jooyoung Lee, Hyun Jung Lee, Seong-Joon Koh, Jong Pil Im, Joo Sung Kim

**Affiliations:** 1 Department of Internal Medicine and Liver Research Institute, Seoul National University College of Medicine, 101 Daehak-ro Jongno-gu, Seoul, 03080 South Korea; 2 Center for Health Promotion and Optimal Aging, Seoul National University Hospital, Seoul, South Korea; 3 Department of Gastroenterology, CHA Bundang Medical Center, CHA University School of Medicine, Seongnam, South Korea; 4 Department of Pathology, College of Medicine, Seoul St. Mary’s Hospital, The Catholic University of Korea, Seoul, South Korea

**Keywords:** Sepsis, Acute lung injury, iRhom2, Macrophage, Multiplex immunohistochemistry

## Abstract

Sepsis has a high mortality rate and leads to multi-organ failure, including lung injury. Inactive rhomboid protease family protein (iRhom2) has been identified as accountable for the release of TNF-α, a crucial mediator in the development of sepsis. This study aimed to evaluate the role of iRhom2 in sepsis and sepsis-induced acute lung injury (ALI). TNF-α and IL-6 secretion in vitro by peritoneal macrophages from wild-type (WT) and iRhom2 knoukout (KO) mice was assessed by enzyme-linked immunosorbent assay. Cecal ligation and puncture (CLP)-induced murine sepsis model was used for in vivo experiments. To evaluate the role of iRhom2 deficiency on survival during sepsis, both WT and iRhom2 KO mice were monitored for 8 consecutive days following the CLP. For histologic and biochemical examination, the mice were killed 18 h after CLP. iRhom2 deficiency improved the survival of mice after CLP. iRhom2 deficiency decreased CD68+ macrophage infiltration in lung tissues. Multiplex immunohistochemistry revealed that the proportion of Ki-67+ CD68+ macrophages was significantly lower in iRhom2 KO mice than that in WT mice after CLP. Moreover, CLP-induced release of TNF-α and IL-6 in the serum were significantly inhibited by iRhom2 deficiency. iRhom2 deficiency reduced NF-kB p65 and IκBα phosphorylation after CLP. iRhom2 deficiency reduces sepsis-related mortality associated with attenuated macrophage infiltration and proliferation in early lung injury. iRhom2 may play a pivotal role in the pathogenesis of sepsis and early stage of sepsis-induced ALI. Thus, iRhom2 may be a potential therapeutic target for the management of sepsis and sepsis-induced ALI.

**Supplementary Information:**

The online version contains supplementary material available at 10.1007/s00418-024-02318-5.

## Introduction

Sepsis, a life-threatening organ dysfunction, arising from an unregulated host response to infection and remains a significant global contributor to mortality (Singer et al. 2016). In 2017, 48.9 million incident cases of sepsis and 11.0 million sepsis-associated fatalities were estimated, accounting for 19.7% of all global death (Rudd et al. 2020). The most important determinant of sepsis prognosis is the occurrence of multiple organ dysfunction syndrome (Costa et al. 2006).

The lungs are the most frequently identified organs that fail to respond to sepsis (Costa et al. 2006). Acute lung injury (ALI) is a critical manifestation in sepsis, which is a severe condition characterized by excessive inflammatory reactions that lead to alveolar injury (Dushianthan et al. 2011). Approximately 30% of sepsis cases involve patients with sepsis-induced ALI, with a mortality rate ranging from 30 to 40% (Gong et al. 2022). Despite intensive research efforts aimed at treating sepsis-induced ALI, supportive lung ventilation remains the only therapy with substantial benefit in terms of mortality (Varisco 2011). Furthermore, early diagnosis of sepsis and lung injury is crucial, as intervention during the reversible stages significantly increases survival in the treatment of these conditions (Rivers et al. 2001).

Tumor necrosis factor-α (TNF-α) plays a key role in systemic inflammatory response by releasing other cytokines in sepsis, and the plasma levels of TNF-α are associated with sepsis-induced death (Georgescu et al. 2020). Macrophages serve as major producers of TNF-α in sepsis and in early stage of sepsis-induced ALI (Kumar 2020; Lee et al. 2021). Although activation and proliferation of macrophages are stimulated by TNF-α in lung inflammation of animal models and human studies, the underlying regulatory mechanism remains elusive.

TNF-α is shed from the plasma membrane subsequent to cleavage by a disintegrin and metalloprotease 17 (ADAM17), an important enzyme accountable for releasing various membrane-anchored substrates, including TNF-α, interleukin-6 (IL-6) receptor, and epidermal growth factor receptor (EGFR) ligands (Black et al. 1997). Inactive rhomboid protease family protein (iRhom2) plays a crucial role in facilitating the forward trafficking of ADAM17 in immune cells (Adrain et al. 2012). Therapeutic blockage of ADAM 17 has multiple side effects on the skin and intestines due to the impairment of EGFR signaling (Calligaris et al. 2021). ADAM17 deficiency in mice is perinatally lethal and causes developmental abnormalities similar to those observed in mice lacking EGFR ligands (Peschon et al. 1998). However, iRhom2 serves as a myeloid-specific regulator of ADAM17 maturation and is particularly enriched in macrophages (Adrain et al. 2012). This underscores that iRhom2 is a promising therapeutic target for TNF-α-dependent diseases, including sepsis. Nevertheless, the role of iRhom2 in macrophages during sepsis and sepsis-induced ALI requires further investigation.

In this study, our objective was to investigate the role of iRhom2 in sepsis and sepsis-induced ALI utilizing a cecal ligation and puncture (CLP) model.

## Materials and methods

### Mice

Wild-type (WT) C57BL/6 mice were obtained from Orient (Seongnam, Korea), while iRhom2 knockout (KO) C57BL/6 mice were sourced from Dr. Tak W Mak (University of Toronto, Toronto, Canada) (Adrain et al. 2012). Male mice were housed in specific pathogen-free conditions at the Center for Animal Resource and Development of Seoul National University (Seoul, Korea). They were provided with standard chow until reaching the desired age (7–8 weeks) as well as body weight (20–24 g). Weekly weighing commenced starting at 3 weeks of age to facilitate comparison of body weight between the two mouse types.

### RNA in situ hybridization and interpretation

To confirm iRhom2 gene KO, RNA in situ hybridization was conducted on formalin-fixed paraffin-embedded lung tissues using the RNAscope FFPE assay kit (Advanced Cell Diagnostics). In brief, 4-μm lung tissue sections from tissue microarrays (TMAs) were pretreated with heat and protease digestion, followed by hybridization with the *Rhbdf2* probe for mouse samples (Advanced Cell Diagnostics, #476,161). Subsequently, a horseradish peroxidase-based signal amplification system was applied, followed by color development with 3,3′-diaminobenzeidine tetrahydrochloride. Positive and negative control slides were simultaneously stained using a mouse PPIB probe (Advanced Cell Diagnostics, #313,911) and bacterial DapB gene probe (Advanced Cell Diagnostics, #310,043), respectively. Nuclei were counterstained with hematoxylin. Positive staining was characterized by brown punctate dots observed in the nucleus and/or cytoplasm.

### Peritoneal macrophages isolation and culture

Peritoneal macrophages from WT and iRhom2 KO mice were harvested following previously described methods (Lee et al. 2014). In brief, peritoneal macrophages were elicited through intraperitoneal injection (2 ml 4% thioglycolate (Thermo Fisher Scientific, #CM0173B) in distilled water). Four days after injection, the elicited macrophages were collected and cultured in 24-well plates (5 × 10^5^ cells/well) for 2 h in 5% CO_2_ at 37 °C. Subsequently, the non-adherent cells were eliminated by phosphate-buffered saline washing, and the attached cells were collected for further in vitro experimentation.

Peritoneal macrophages obtained from both WT and iRhom2 KO mice were stimulated with lipopolysaccharide (LPS) from *Eschericia coli* 0127:B8 (Sigma-Aldrich, #L3129, 1 µg/ml LPS for 4 h), a Toll-like receptor 4 ligand known for its potent inflammatory-inducing properties and found in the cell wall of gram-negative bacteria. Following LPS stimulation, TNF-α and IL-6 concentration in the culture supernatants was determined using commercially available enzyme-linked immunosorbent assay (ELISA) kit (R&D Systems, #MTA00B and #M6000B).

### Cecal ligation and puncture (CLP) model

CLP was conducted following previously reported procedures with small adjustments (Rittirsch et al. 2009). Upon induction of anesthesia with isoflurane (2–3%), the surgical area was sterilized with 70% alcohol, and a midline laparotomy was performed. The cecum that was identified during the procedure was ligated in the distal 75% portion with 5–0 ethilon suture (Ethicon, #1666G). This was followed by a single pass puncture with a 23-gauge needle (Becton Dickinson, #305,145). Closure of the peritoneal cavity was achieved using 6–0 nylon sutures (AD surgical, #S-N618XR13). After the operation, sterile normal saline (0.5 ml) was administered via intraperitoneal injection. Identical procedures were done for sham laparotomy controls, including opening of the peritoneum and exposing the bowel, but CLP was excluded. Subsequently, the mice were killed 18 h after CLP for histologic and biochemical examination.

### Survival study

All CLP mice were monitored for 8 consecutive days to evaluate the impact of iRhom2 deficiency on their survival. Surviving mice were killed 8 days after CLP.

### Immunohistochemistry

Lung tissue specimens were obtained from WT sham, KO sham (*n* = 4 per group for both), CLP WT, and CLP iRhom2 KO (*n* = 8 per group for both) mice. They were fixed in a buffered 10% formalin solution and embedded in paraffin. To prepare TMAs, representative 4-mm cores from each donor block were rearranged into new recipient blocks using a trephine apparatus (SuperBiochips Laboratories). After preparing 4-μm-thick sections and deparaffinizing, antigen retrieval was conducted with EDTA buffer (pH = 9.0) for 15 min at 100º C followed by peroxidase blocking with H_2_O_2_.

The slides were stained with antibodies including those against myeloperoxidase (MPO) (rabbit polyclonal antibody IgG, Dako, # A0398, 1:200), CD68 (rabbit monoclonal antibody IgG, Cell Signaling Technology, #97778S, 1:150), CD3 (rabbit monoclonal antibody IgG, Novus Biologicals, # NB600-1441, 1:100), Ki-67 (rabbit polyclonal antibody IgG, Abcam, #ab15580, 1: 400), phospho-NF-κB p65 (Ser 536) (rabbit polyclonal antibody IgG, Santa Cruz Biotechnology, #sc-33020, 1:100), and IκBα phospho (ser32/Ser36) (rabbit polyclonal antibody IgG, Arigo Biolaboratories, # ARG51651, 1:100). The slides were then exposed to dextran polymer coupled with anti-rabbit IgG and horseradish peroxidase (Dako, #K4063). For chromogenic reactions and visualization, peroxidase detection was performed with deaminpobenzidine solution (Dako, #K3468).

The open‐source software QuPath was used (https://qupath.github.io/) to quantify the MPO+ neutrophils, CD68+ macrophages, CD3+ T cells, and Ki-67+ cells. Cell density was calculated as the number of cells in a given area (mm^2^), and the mean density was used for statistical analysis.

To investigate the proliferative activity of CD68 + macrophages and CD3+ T cells, multiplex immunohistochemistry (mIHC) was performed by consecutive staining of CD68, CD3, and Ki-67 on the same slide using Autostainer Link48 (Dako) (Koh et al. 2020). Initially, hematoxylin staining and whole-slide image scan were conducted to identify the nuclei. Subsequently, three cycles of IHC were performed involving antigen retrieval, incubation with primary antibodies (CD68, CD3, and Ki-67), secondary reagent incubation, chromogenic reaction using ImmPact AEC (3-amino-9-ethylcarbazole) substrate (Vector Laboratories), whole-slide image scan, and stripping. The stripping solution (β-mercaptoethanol in SDS buffer) removed the chromogen as well as antibodies. The density of Ki-67+ CD3+ , ki-67+ CD68+ , CD3+, and CD68+ cells were quantified separately using the CellProfiler image analysis program (ver. 3.1.8, Broad Institute), and the percentages of double-positive cells among CD3+ cells and CD68+ cells were calculated, respectively. Detailed methodologies can be referenced in the previous publication (Koh et al. 2020).

### TUNEL assay

The terminal deoxynucleotidyl transferase dUTP nick end labeling (TUNEL) assay was performed on lung tissues (Koh et al. 2011) to detect cell apoptosis using the ApopTag + detection system (Millipore). The density of apoptotic cells was determined by counting the number of TUNEL-positive cells in a given area (mm^2^) using CellProfiler image analyzer program (ver 3.1.8, Broad Institute).

### Measurement of cytokines in the serum by ELISA

The levels of TNF-α and IL-6 in the serum were measured using ELISA kits according to the manufacturer’s instructions (R&D systems).

### Ethical considerations

All animal procedures were approved by the Institutional Animal Care and Use Committee of Seoul National University (IACUC no. SNU-170404–21). All animal experiments were carried out in accordance with the relevant guidelines, regulations, and ARRIVE guidelines (Kilkenny et al. 2010).

### Statistical analyses

Data were presented as mean ± standard deviation, and statistical analyses were carried out using GraphPad Prism software, version 9.1 (GraphPad). Student’s t-test or one-way analysis of variance (ANOVA) was employed for data analysis. Kaplan-Meier survival curves were assessed using the log-rank test. A *p*-value < 0.05 was considered as statistically significant.

## Results

### Knock-out iRhom2 RNA expression is measured by RNA in situ analysis

**Fig. 1:**
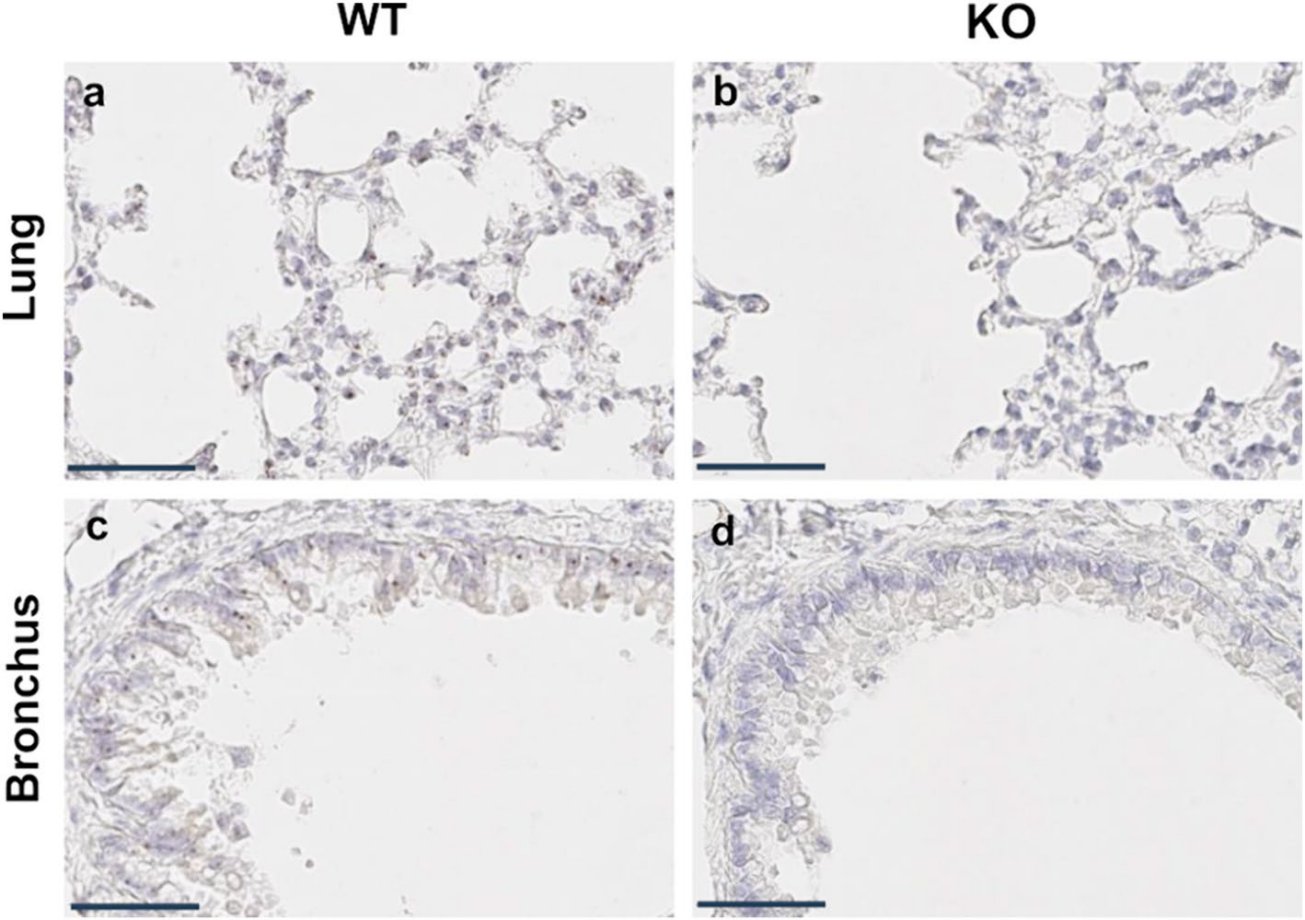
mRNA in situ hybridization of iRhom2 using RNAscope probe sets (scale bar = 50 μm). **a** Lung parenchyma of wild-type (WT) mouse. **b** Lung parenchyma of knockout (KO) mouse. **c** Bronchus of WT mouse. **d** Bronchus of KO mouse. WT mice showed brown-colored iRhom2 mRNA signals in the nucleus and cytoplasm, whereas KO mice did not show any signals

RNA in situ analysis of iRhom2 mRNA, as depicted in Fig. 1, confirmed the KO of the iRhom2 gene in the lung tissues of the experimental models. iRhom2 mRNA signals were detected in the nucleus and cytoplasm of cells in the lung parenchyma (Fig. 1a) and bronchus (Fig. 1c) of WT mice but were not detected in either the lung parenchyma (Fig. 1b) or bronchus (Fig. 1d) of iRhom2 KO mice.

### iRhom2 deficiency inhibits secretion of inflammatory cytokine TNF-α in lipopolysaccharide-stimulated peritoneal macrophages

**Fig. 2:**
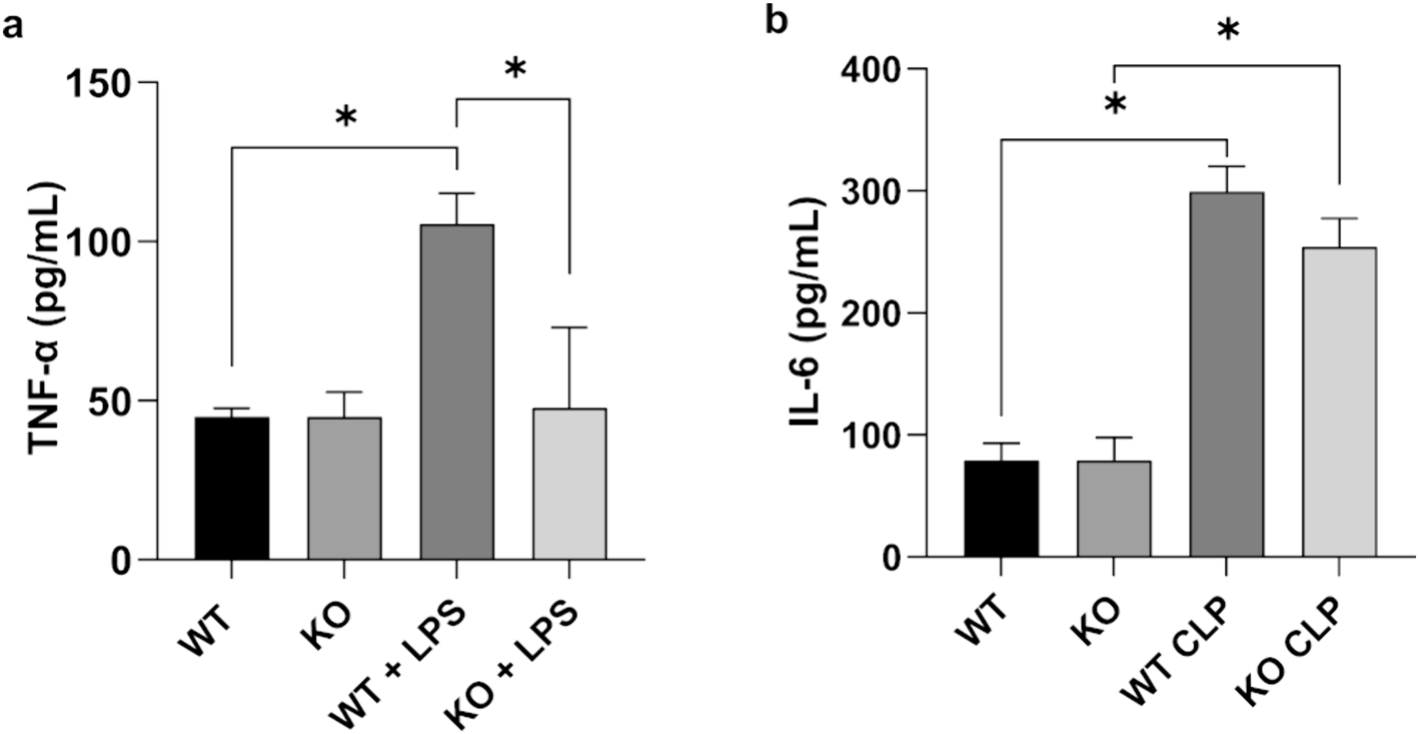
Effect of iRhom2 deficiency on inflammatory cytokine secretion. Peritoneal macrophages from WT mice and iRhom2 KO mice were stimulated with LPS (1ug/ml LPS for 4 h). **a** TNF-α and **b** IL-6 secretion in the culture supernatants were measured by ELISA. LPS-induced TNF-α secretion by macrophages was significantly inhibited by iRhom2 deficiency. *p < 0.05

We compared TNF-α and IL-6 secretion in vitro by peritoneal macrophages from WT and iRhom2 KO mice by using ELISA assay. In WT macrophages, the secretion of TNF-α and IL-6 was markedly increased after LPS treatment. In contrast, in iRhom2 KO macrophages, only IL-6 secretion exhibited a significant increase after LPS treatment, while TNF-α secretion remained unchanged. The LPS-induced secretion of TNF-α in peritoneal macrophages was significantly inhibited by iRhom2 deficiency (Fig. 2a, p = 0.02). No significant differences were observed in the LPS-induced secretion of IL-6 between WT and iRhom2 KO macrophages (Fig. 2b, p = 0.07). Therefore, our results indicate that iRhom2 deficiency mitigates the proinflammatory cascade in macrophages by inhibiting LPS-induced TNF-α secretion.

## iRhom2 deficiency enhances survival in mice with sepsis induced by CLP

**Fig. 3:**
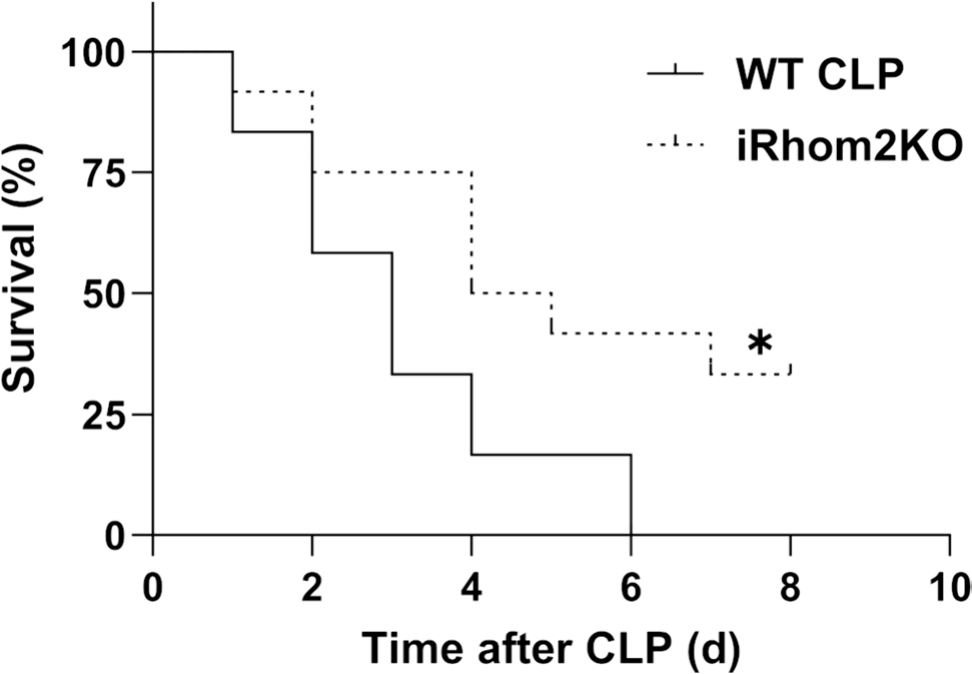
Effect of iRhom2 deficiency on survival rates after CLP. WT and iRhom2 KO mice (*n* = 12 per group) underwent CLP, and their survival was monitored for 8 days. Survival rates between the two groups were analyzed using Kaplan-Meier analysis and log-rank tests. *p < 0.05

Untainted iRhom2 KO mice were viable and exhibited overall good healthy, consistent with findings from previous studies (Supplementary Fig. S1) (Adrain et al. 2012). To evaluate the role of iRhom2 deficiency on survival during sepsis, both WT and iRhom2 KO mice (*n* = 12 per group) were monitored for 8 consecutive days following the CLP procedure. In the WT mice, survival rate decreased to 58.3% (7/12) on the 2nd day and to 16.7% (2/12) on the 4th day. In the iRhom2 KO mice, survival rates decreased to 75.0% (9/12) on the 2nd day and to 50.0% (6/12) on the 4th day. iRhom2 KO mice showed a significant advantage in survival after CLP (*p* = 0.01, Fig. 3).

### iRhom2 deficiency reduces infiltration of macrophages in the lung tissues after CLP

**Fig. 4:**
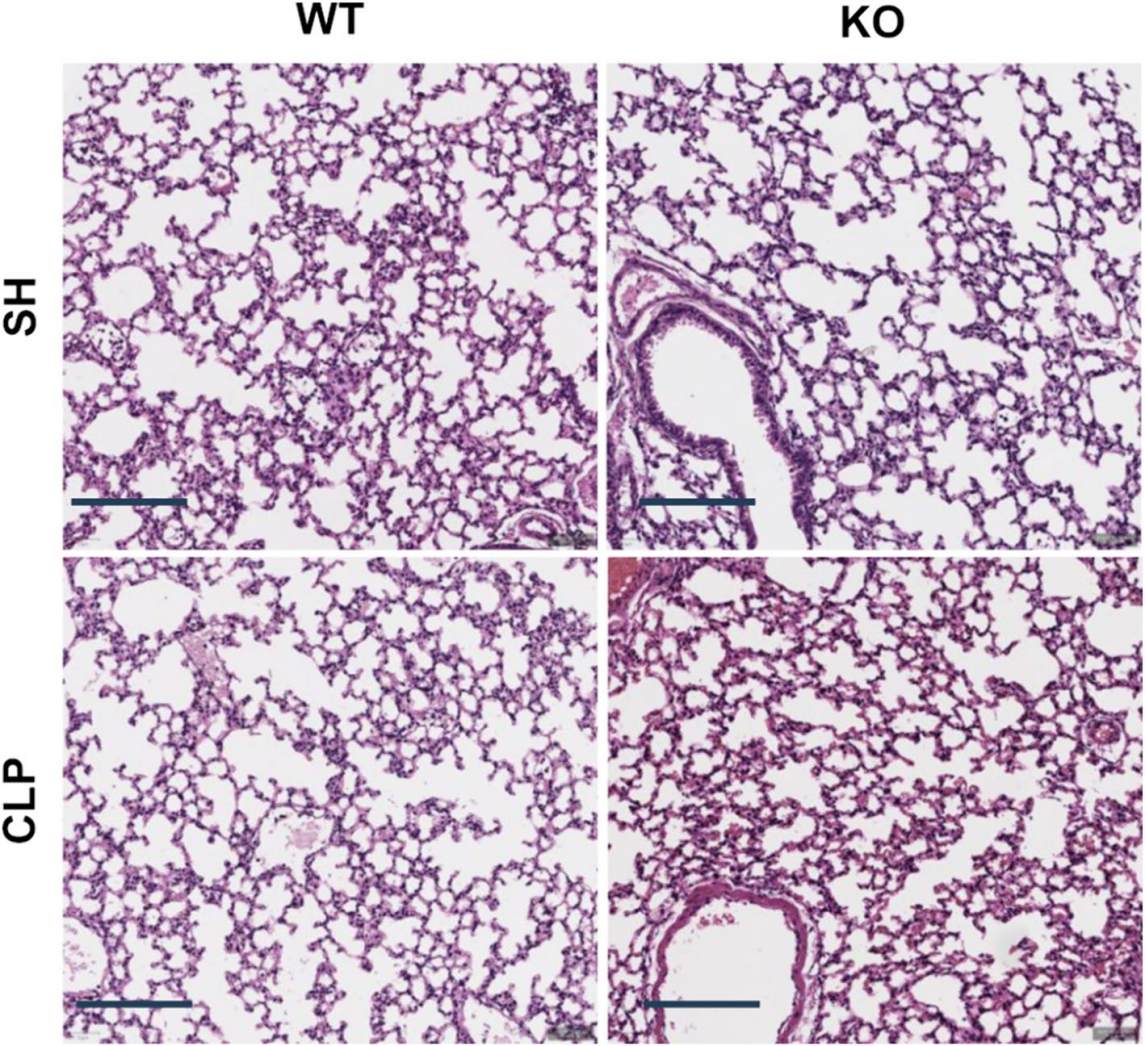
Effect of iRhom2 deficiency on histologic changes in lung tissue after CLP. Representative histologic sections of hematoxylin and eosin (H&E)-stained lung tissues from WT and iRhom2 KO mice are shown (scale bar = 200 μm). WT and iRhom2 KO mice were randomized into a sham laparotomy group (*n* = 4 each) and CLP group (*n* = 8 each) as follows: WT SH (sham-operated WT group), KO SH (sham-operated KO group), WT CLP (CLP-operated WT group), and KO CLP (CLP-operated KO group)

**Fig. 5:**
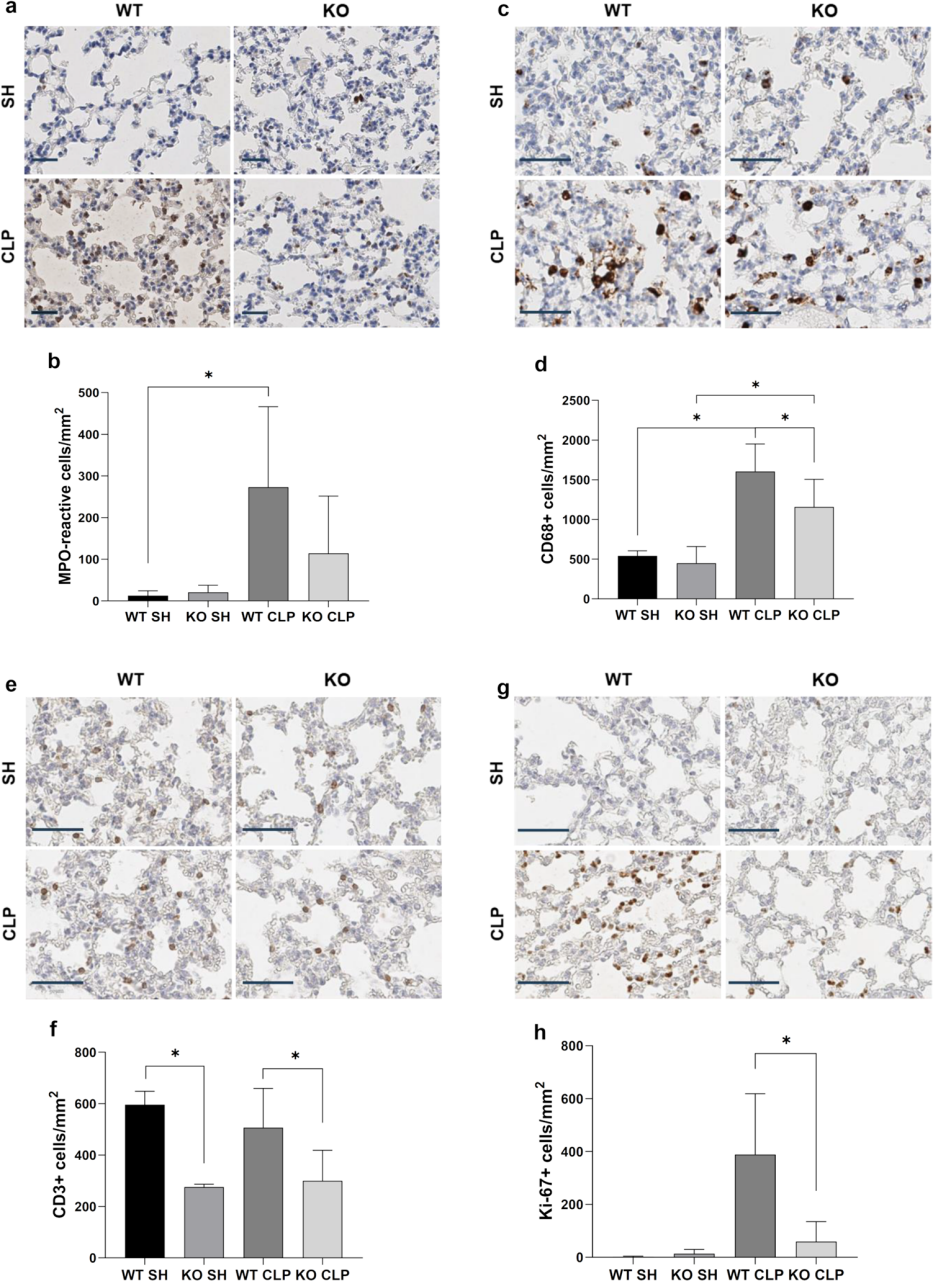
Histology and quantified density of MPO+ neutrophils (**a** and **b**, respectively), CD68 + macrophages (**c** and **d**, respectively), CD3+ T cells (**e** and **f**, respectively), and Ki-67 cells (**g** and **h**, respectively) in lung tissues (scale bar = 50 μm). iRhom2 deficiency reduced CD68 + macrophage infiltration of lung tissues after CLP. Lung tissues were collected at 18 h after CLP or sham operation. *p < 0.05

The results of H&E staining of the lung tissue microarray slides are shown in Fig. 4. No significant histologic differences were observed between the sham and CLP groups in either WT or KO mice 18 h after CLP (data not shown) in terms of the lung injury score. Subsequent IHC for the neutrophil marker MPO, macrophage marker CD68, and pan-T cell marker CD3 was performed on lung TMA slides to detect changes in the infiltration of these immune cells in the early stage of sepsis-induced ALI before histologic damage was observed (Fig. 5a, c, and e, respectively).

The density of neutrophils in lung tissues of WT mice after CLP was higher than that of iRhom2 KO mice; however, this difference was statistically insignificant (*p* = 0.08, Fig. 5b).

Statistical analyses suggested that macrophage density was not different in the sham-operated group between WT and iRhom KO mice but increased after CLP operation (Fig. 5d). In contrast, macrophage density was significantly lower in iRhom2 KO CLP mice than in WT CLP mice (*p* = 0.03). Our results indicate that iRhom2 deficiency affects macrophage density in the CLP group but not in the sham-operated group.

Since previous findings indicated that T cells are potent early mediators of the host response to sepsis (Kasten et al. 2010), we hypothesized that iRhom2 deficiency could also affect T cell density in our specimens. IHC for CD3 (Fig. 5e) followed by statistical analyses showed that CLP did not alter T cell density (Fig. 5f), whereas iRhom2 deficiency induced a significant decrease in T cell density in both groups (*p* < 0.01). In summary, our results showed that iRhom2 deficiency significantly decreased the infiltration of macrophages, but not of neutrophils and T cells, in lung tissue specimens obtained from the early stage of sepsis-induced ALI before histologic damage was observed.

### iRhom2 deficiency reduces the proliferation of macrophages but not that of T cells after CLP

To elucidate the mechanism underlying the effect of iRhom2 on immune cell density in sepsis-induced ALI, we examined the proliferative activity of CD68 + macrophages and CD3+ T cells. First, we performed IHC for Ki-67 (Fig. 5g), which revealed that iRhom2 deficiency induced significant reduction in nuclear Ki-67+ localization compared to WT mice after CLP (*p* < 0.01, Fig. 5h).

**Fig. 6:**
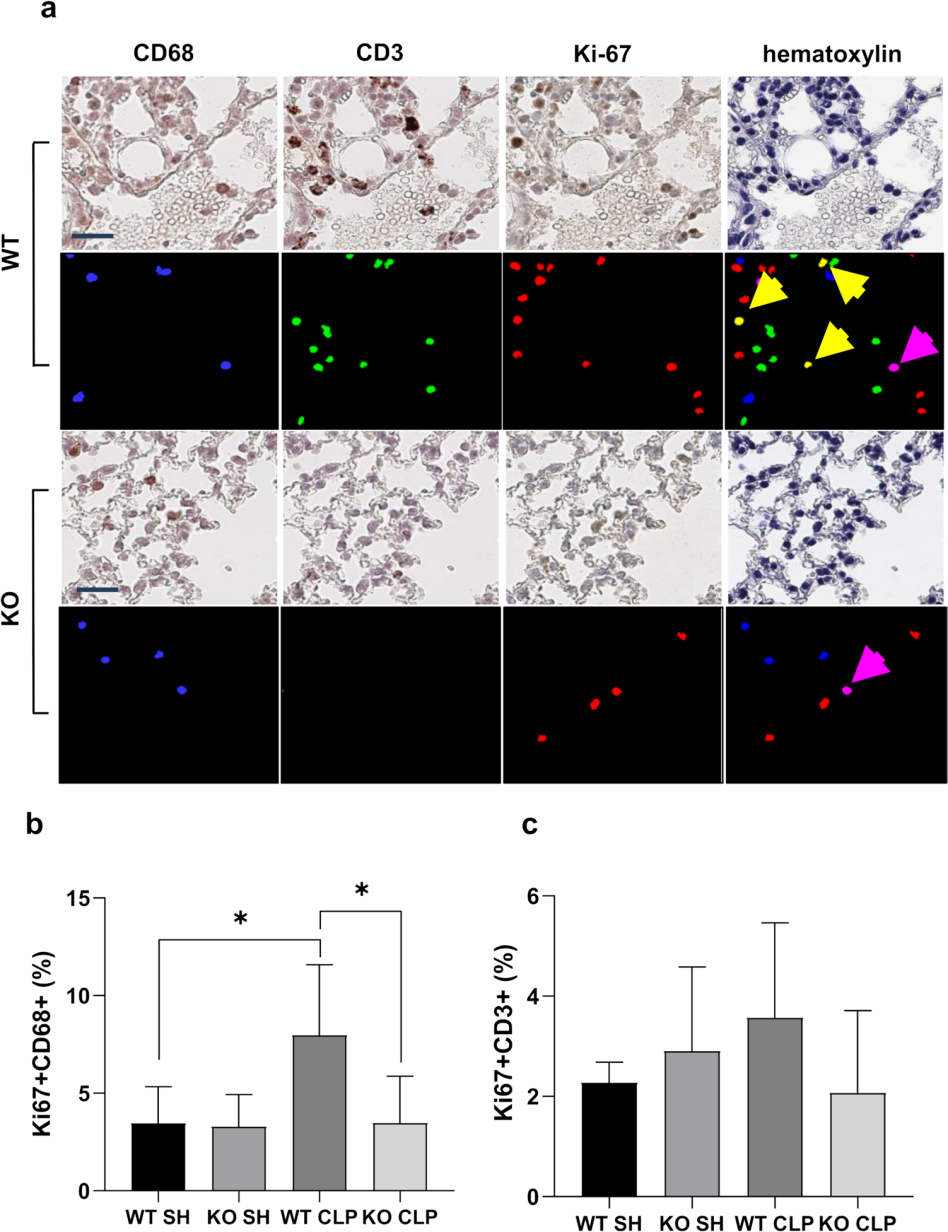
Effect of iRhom2 deficiency on the proliferation of the immune cells in lung tissue in mice after CLP. **a** Multiplex IHC of lung parenchyma in WT mice and iRhom2 KO mice. A single slide was subsequently stained with CD68, CD3, and Ki67 and assigned pseudo-colors as follows: CD 68 as blue, CD3 as green, and Ki67 as red. Ki67^+^CD68^+^ macrophages (yellow arrows) and Ki67+ CD3+ T cells (Magenta arrows) can be identified in pseudo-color image (scale bar = 25 μm). **b** The proportion of Ki-67+ macrophages in total CD 68+ macrophages was significantly decreased in the iRhom2 KO mice compared with the WT mice after CLP. **c** The proportion of Ki-67+ T cells in total CD3+ T cells was not affected by iRhom2 KO in either sham-operated group or CLP group. *p < 0.05

We then identified the source of Ki-67+ cells via mIHC, which localized macrophages, T cells, and all Ki-67+ cells in identical sections from WT and iRhom2 KO mice (Fig. 6a). mIHC can intuitively visualize immune cells by assigning pseudo-colors to each combination of positive markers. Ki67+ CD68+ macrophages and Ki67+ CD3+ T cells can be identified in pseudo-color image as yellow and magenta, respectively. CD68, CD3, Ki67+ cells, and Ki67+ CD68+ macrophages in the lung tissue were reduced in iRhom2 KO CLP mice compared to those in WT CLP mice (Fig. 6a). Then, we evaluated the proportions of Ki-67+ cells among the macrophages (Fig. 6b) and T cells (Fig. 6c). The proportion of Ki-67+ macrophages was significantly higher in WT CLP mice than in WT sham mice (*p* = 0.04). iRhom2 deficiency did not induce significant change in the macrophage proliferation in the sham-operated group (*p* = 0.70), whereas a 57% decrease was observed in the CLP group (*p* = 0.02, Fig. 6b). These results indicate that iRhom2 deficiency affects macrophage proliferation in the CLP group but not in the sham-operated group.

In contrast, iRhom2-deficiency did not significantly affect T cell proliferation in either the sham-operated group (*p* = 0.32) or the CLP group (*p* = 0.13, Fig. 6c). These data suggested that iRhom2 deficiency contributes to decrease the macrophage infiltration by decreasing cell proliferation in sepsis-induced ALI.

### iRhom2 deficiency reduced systemic release of inflammatory cytokine after CLP

**Fig. 7:**
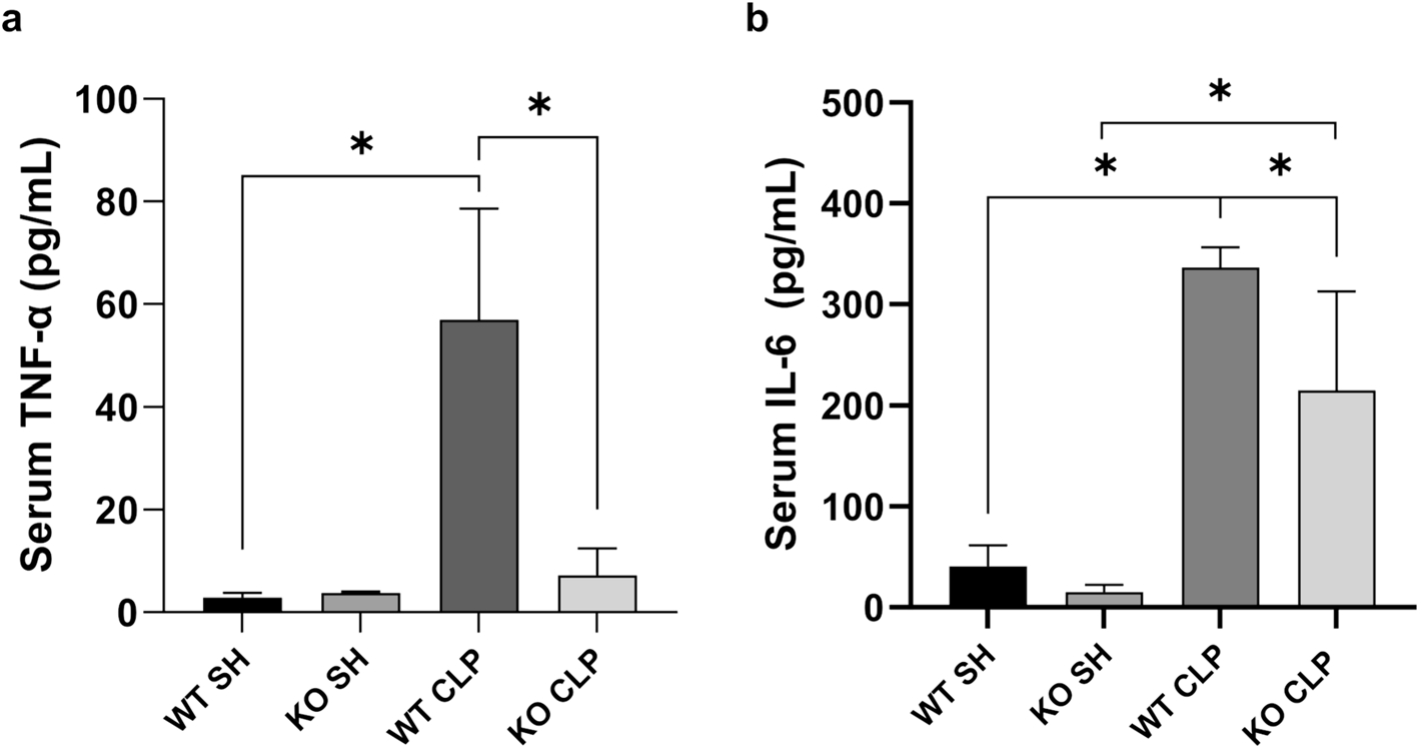
Effect of iRhom2 deficiency on the release of inflammatory cytokines after CLP. **a** TNF-a and **b** IL-6 in the serum were measured by ELISA. CLP-induced release of TNF-α and IL-6 in the serum were significantly inhibited by iRhom2 deficiency. *p < 0.05

To evaluate the effect of iRhom2 deficiency on the systemic inflammatory response following CLP, we measured the serum levels of two inflammatory cytokines, TNF-α and IL-6 (Fig. 7a and b). In the WT mice, serum levels of TNF-α and IL-6 were markedly increased after CLP. However, in the KO mice, only serum level of IL-6 significantly increased after CLP, while the level of TNF-α remained unchanged. iRhom2 deficiency significantly inhibited the CLP-induced release of both TNF-α and IL-6 in the serum (both *p* < 0.01).

### iRhom2 deficiency does not affect the apoptosis after CLP

**Fig. 8:**
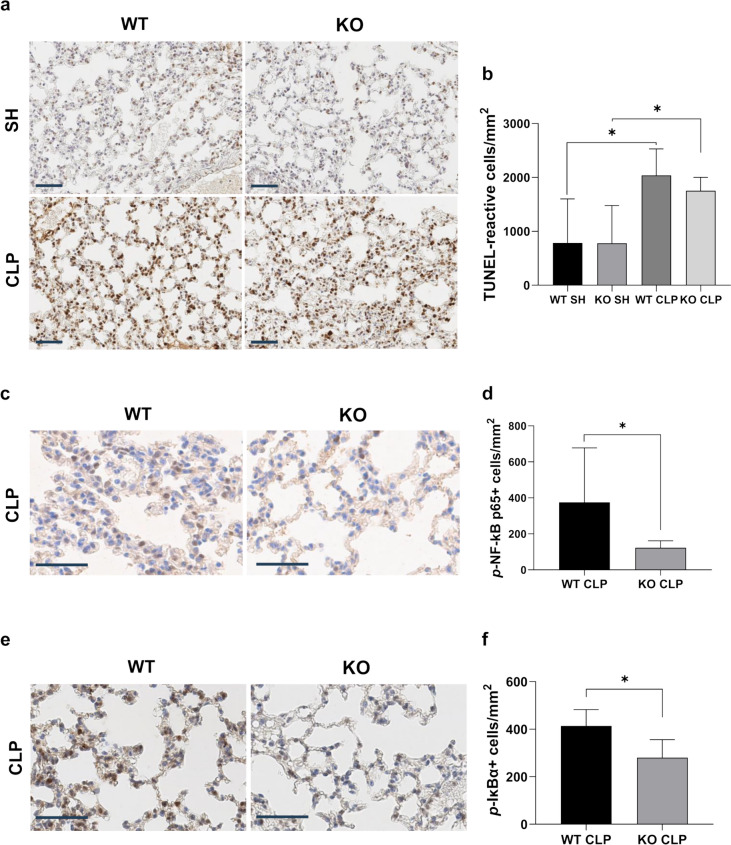
TUNEL and the expression of phospho-NF-kB p65 and phospho-IκBα in WT and KO mice. **a** Representative histologic sections of TUNEL staining on lung tissue from WT mice and iRhom2 KO mice are shown (scale bar = 50 μm). **b** Digitally quantified TUNEL-reactive cell density of lung tissues was not significantly different in the KO mice compared with the WT mice after CLP. **c** Representative images of phosphorylated NF-kB p65 immunohistochemical staining of lung tissues in after CLP. iRhom2 deficiency attenuated the degree of phosphorylated NF-kB p65 staining in the lung tissue after CLP. **d** Digitally quantified phosphorylated NF-kB p65 positive cell density of lung tissues was significantly decreased in the iRhom2 KO mice compared with the WT mice after CLP. **e** Representative images of phosphorylated IκBα immunohistochemical staining of lung tissues in after CLP. iRhom2 deficiency attenuated the degree of phosphorylated IκBα staining in the lung tissue after CLP. **f** Digitally quantified phosphorylated IκBα positive cell density of lung tissues was significantly decreased in the iRhom2 KO mice compared with the WT mice after CLP. *p < 0.05

TUNEL staining was conducted to assess the impact of iRhom2 deficiency on apoptosis in the lung tissues (Fig. 8a). TUNEL staining confirmed positive staining throughout the cells including alveolar and immune cells in alveolar part of the lung. TUNEL-reactive cell density significantly increased after CLP (*p* = 0.01). Meanwhile, iRhom2 deficiency did not result in significant difference in the density of TUNEL-reactive cells between iRhom2 KO and WT CLP mice (*p* = 0.19, Fig. 8b).

### iRhom2 deficiency decreases NF-κB signaling after CLP

NF-κB is a key player in transcriptional induction of proinflammatory mediators, and its activation is considered a significant pathologic mechanism underlying septic shock and inflammation (Liu and Malik 2006). Hence, we explored the effect of iRhom2 deficiency on the NF-κB signaling pathway. The expression levels of phospho-NF-kB p65 and phospho-IκBα, indicative of active NF-kB, were assessed in WT and iRhom2 KO mice via IHC staining. IHC analysis demonstrated that phosphorylation of NF-kB p65 and IκBα was significantly inhibited in iRhom2 KO mice undergoing CLP compared with the WT mice (Fig. 8c–f). Morphologic analysis revealed staining across various cell types, including alveolar and immune cells.

## Discussion

Using a CLP-induced murine sepsis model, we demonstrated that iRhom2 exerts a significant influence on sepsis and early stages of sepsis-induced ALI. iRhom2 deficiency reduces mortality after CLP and decreases CD68+ macrophage infiltration and proliferation in the early stage of sepsis-induced ALI. In contrast, iRhom2 deficiency did not affect the infiltration and proliferation of CD3+ T cells in the lung tissues after CLP. Moreover, iRhom2 KO mice showed decreased NF-κB signaling in the lung tissues after CLP compared to WT mice. To our knowledge, this study represents the first investigate into the role of iRhom2 in sepsis and sepsis-induced ALI.

TNF‐α modulates cellular functions such as cell proliferation, survival, differentiation, and apoptosis, which has been well known to initiate sepsis (Georgescu et al. 2020). However, several clinical trials failed to show a survival gain for anti-TNF-α treatment in patients with sepsis (Marshall 2014). It has been suggested that elevated TNF-α could be a non-causal associate of sepsis and that insufficient TNF-α blockade may account for the failure of anti-TNF-α treatment (Gharamti et al. 2022). Given that TNF-α produced by macrophages plays a pivotal role in the infiltration of immune cells into damaged organs during sepsis, the need to selectively block TNF-α secreted by macrophages has been suggested (Lee et al. 2021). iRhom2 is predominantly expressed in macrophages and is upregulated in response to LPS stimulation (Adrain et al. 2012). Therefore, we evaluated iRhom2 as a putative therapeutic target in sepsis.

The CLP model stands as the most extensively employed model for experimental sepsis and is considered the gold standard in research due to its ability to mimic the progression of severe sepsis observed in humans (Hubbard et al. 2005). Although iRhom2 has been studied in various diseases, including inflammatory and immune responses, its specific role in reducing mortality in sepsis has not been well established (McIlwain et al. 2012; Lee et al. 2021). iRhom2 KO mice showed increased survival at lethal doses of LPS but were more susceptible to *Listeria monocytogenes* infection than WT mice (McIlwain et al. 2012). A previous study reported that small interfering RNA-silencing ADAM17 in macrophages improves survival rates in septic mice (Lee et al. 2021). In the present study, iRhom2 KO mice exhibited a significant improvement in survival after CLP. Thus, we speculated that iRhom2 manipulation could be clinically useful for treating patients with sepsis.

In our current study, H&E staining of the lung tissue showed no obvious histologic changes after CLP, suggesting that our model reflects the early stage of sepsis-induced ALI. CLP is known to cause mild lung injury akin to ALI, albeit with less pronounced intra-alveolar inflammation and hyaline membrane formation (Matute-Bello et al. 2008). Previous studies have demonstrated a substantial rise in the histologic injury score in the lung tissues of the CLP group compared to the sham-operated group (Matute-Bello et al. 2008; Li et al. 2013). The difference between studies in terms of histologic injury can be explained by the degree of induced sepsis and duration of sepsis before sacrifice. Because CLP-induced sepsis has a high mortality rate, to evaluate the development of early stage of sepsis-induced ALI, we killed mice within 20 h, which was known as the therapeutic window in previous studies. The therapeutic window period for sepsis is important because beneficial interventions are available during this reversible phase (Cauvi et al. 2012). In addition, our study used a modified CLP protocol to induce mild sepsis compared with previous studies (Hirano et al. 2015; Li et al. 2013; Rittirsch et al. 2009). Although histologic changes in sepsis induced-ALI were not directly confirmed, given the similar mortality rate observed in previous ALI studies using the CLP procedure (Li et al. 2013) and increased systemic release of inflammatory cytokine after CLP, it can be assumed that sepsis-induced ALI was adequately established in this study.

LPS represents the most prevalent component within the cell wall of gram-negative bacteria. Exposure to LPS induces ADAM17 activity through rapid transcriptional upregulation of iRhom2 in macrophages (Adrain et al. 2012). Several studies have demonstrated that iRhom2 KO macrophages exhibit impaired TNF-α secretion in response to LPS (Sweet and Hume 1996; McIlwain et al. 2012). In this study, we performed an in vitro study to confirm the previous reports by using peritoneal macrophages obtained from WT and iRhom2 KO mice. Consistent with previous reports, TNF-α secretion upon LPS treatment was significantly reduced in the iRhom2 KO macrophages compared to WT macrophages. However, the increase in TNF-α secretion after LPS treatment observed in our study was modest compared to the previous study (McIlwain et al. 2012). This discrepancy may be attributable to variations in the duration of LPS treatment across studies. According to another study investigating the effects of LPS treatment on macrophages, TNF-α secretion by macrophages increased rapidly during 4–8 h after LPS treatment, with no continuous increase observed thereafter (Reis et al. 2011). In the aforementioned study (McIlwain et al. 2012), TNF-α in culture supernatants was measured 24 h after LPS treatment, whereas in our study, TNF-α was assessed at 4 h after LPS treatment.

We also observed that LPS-induced IL-6 secretion by peritoneal macrophages was unaffected by iRhom2 deficiency, consistent with prior study findings (Adrain et al. 2012; Kim et al. 2018; McIlwain et al. 2012). However, our in vivo study reveals that the release of both TNF-α and IL-6 into the serum was significantly diminished by iRhom2 deficiency, although the impact on IL-6 secretion was not as pronounced as that on TNF- α. The effect of iRhom2 deficiency on IL-6 secretion in inflammatory disease has been reported to be variable and context-dependent (Chenxu et al. 2018; McIlwain et al. 2012; Qing et al. 2018). IL-6 production is triggered by EGFR stimulation, as well as TNF- α, whose ligand is also cleaved by ADAM 17. In addition, IL-6 receptor (IL-6R), an ADAM17 substrate that mediates the IL-6 signaling pathway, binds to IL-6 in its soluble form and also influences the serum level of IL-6 (Schumacher and Rose-John 2019). Another study revealed a relatively modest effect of ADAM17 deficiency on soluble IL-6R release, suggesting that alternative protease may contribute to IL-6R processing under inflammatory conditions (Schumacher et al. 2016). In addition to macrophages, epithelial cells are also recognized for their role in producing IL-6 during lung injury (Okuma et al. 2023; Quinton et al. 2008). This complexity in influencing systemic levels of IL-6 may account for the variable results observed between studies and the discrepancies between in vitro and in vivo findings related to IL-6 in this study.

Neutrophils are known as the first immune cells to infiltration during lung injury. In a previous study, CLP led to an elevation of neutrophils in the bronchoalveolar compartment of the lung starting from 1 h after CLP. This effect persisted throughout the 18-h observation period (Razavi et al. 2004). Another study showed that alveolar macrophages played an important role in the recruitment of neutrophils very early in the course of LPS-induced lung injury. However, at a later time point during LPS-induced lung inflammation, no disparity in neutrophil recruitment was noted between lungs with or without alveolar macrophages (Beck-Schimmer et al. 2005). In the present study, we observed that iRhom2 deficiency decreased the infiltration of MPO + neutrophils in the lung tissues after CLP; however, this effect was statistically insignificant. This difference in neutrophil infiltration between the two groups could be more clearly identified if the assessments were made at a very early stage of lung injury.

Macrophages are vital for the regulation of innate immunity and host defense in the lungs. Several studies support the perspective that macrophages not only initiate and maintain the inflammatory response but also contribute to the resolution of lung inflammation (Kumar 2020). In CLP-induced sepsis, macrophages are activated in response to invading bacteria and bacterial products that escape from the punctured cecum. Studies have revealed that the survival of macrophages is dependent on autocrine signaling by TNF-α (Wolf et al. 2017; Lombardo et al. 2007). Since TNF-α orchestrates numerous pathologic effects observed in septic shock, it is suggested that sustained macrophage survival mediated by TNF-α is essential in sepsis.

In the current study, IHC analysis demonstrated that iRhom2 KO decreased macrophage infiltration, which is consistent with the results of in vitro study, and suggests that deficiency of iRhom2 exhibits a protective role in lung inflammation. However, it remains unclear whether the increase in macrophage infiltration is caused by an increase in cell proliferation. Thus, we utilized mIHC, a novel method of sequentially staining IHC markers on a single FFPE tissue slide for the co-visualization of the macrophage marker CD68 and the proliferation marker Ki-67 to demonstrate that iRhom2 deficiency reduced the proliferation of macrophages in the CLP group, but not in the sham-operated group. mIHC is an effective and efficient method for concurrently identifying specific proteins or molecular abnormalities as well as determining the activation state of immune cells and presence of the immunoactive molecular expression (Koh et al. 2020). This technique facilitates the simultaneous analysis of multiple markers within a single FFPE tissue section, offering accurate cell discrimination and spatial information (Son et al. 2020). Remark et al. proved that multiple cycles of mIHC do not decrease antigenicity or cause steric hindrance. The density of tumor-associated immune cells for various markers remained consistent across multiple destaining cycles of mIHC, from one to seven (Remark et al. 2016).

The interaction between macrophages and other immune cells, including neutrophils and T cells, is pivotal in influencing the outcome of CLP-induced sepsis. T cells, in particular, emerge as potent early mediators of the host response to sepsis. TNF-α can stimulate proliferation and activation of T cells, while also inducing apoptosis of activated effector T cells, which determines the size of the pathogenic or protective conventional T cell pool (Kasten et al. 2010). Recently, it was reported that alterations in T cell subtypes also play an important role in the pathophysiology of sepsis-induced ALI. Our findings suggest that CLP does not influence either infiltration or proliferation of T cells. However, iRhom2 deficiency significantly decreased T cell infiltration in both the sham and CLP groups. iRhom2 may affect T cells through several potential mechanisms such as cytokine regulation, T cell receptor signaling, and T cell migration (Link et al. 2017). Considering that iRhom2 had no effect on the proliferation of T cells in either the sham or CLP groups, the effect of iRhom2 on the infiltration of T cells could be due to a process other than proliferation, such as T cell recruitment.

Enhanced apoptosis of cells in the lung tissues, including alveolar and airway epithelial cells and endothelial cells, has been suggested as additional potential mechanism contributing to ALI. Macrophages engulf apoptotic neutrophils, which may modulate neutrophil-mediated lung injury (Fan and Fan 2018). Several studies have reported that the therapeutic regulation of macrophages attenuates ALI through its effects on pulmonary parenchymal apoptosis (Kishta et al. 2012; Fan and Fan 2018). According to a previous study, iRhom2 deficiency results in decreased apoptosis in ischemia-reperfusion-mediated ALI (Kim et al. 2018). However, in our study, iRhom2 deficiency did not significantly affect cell apoptosis in either sham or CLP groups. The difference in these results can be explained by the fact that the lung injury induced in this study was mild compared to that in previous studies.

TNF-α signaling is known to activate the NF-κB signaling pathway, which encompasses various genes promoting cell survival and inhibiting cell death by apoptosis (Karin and Lin 2002). NF-κB is a ubiquitous transcription factor expressed in most types of cells, including alveolar and immune cells (Perkins and Gilmore 2006). NF-κB is bound by an inhibitory molecule, IκBα. When inflammatory cascade is triggered, phosphorylation of IκBα increases, resulting in the increased degradation of IκBα. The transcriptional activity of NF-kB is also regulated by the phosphorylation of p65 subunit at Ser536 by IkB kinases (Sakurai et al. 2003). Thus, phospho-NF-kB p65 and phospho-IkB are indicators of NF-kB activation. NF-κB pathway is crucial in the pathogenesis of ALI. A recent study in a rat model of LPS-induced ALI revealed that inhibition of the Toll-like receptor 4/NF-κB signaling pathway reduces oxidative stress, thereby alleviating ALI (Zhang et al. 2019). Activation of NF‐κB in alveolar macrophages is known to be important in initiating lung inflammation. These macrophages serve as the first responders, with NF‐κB activation leading to the production of cytokine that subsequently activate NF‐κB in other cells (Alvira 2014). In this study, the analysis of NF‐κB activation status, determined by IHC staining in lung tissues after CLP, revealed that NF‐κB activity is abolished by iRhom2 deficiency. This result suggests that iRhom2 deficiency decreased macrophage infiltration and proliferation, at least in part, by inhibiting NF-κB signaling pathways.

In conclusion, iRhom2 deficiency reduces sepsis-related mortality and is associated with decreased macrophage infiltration and proliferation in early lung injury. These findings suggest iRhom2 could serve as a novel therapeutic target for sepsis.

## Supplementary Information

Below is the link to the electronic supplementary material.

Supplementary file1 (TIF 39 KB)

## Data Availability

No datasets were generated or analysed during the current study.
