# Supplementary figures and images for: iRhom2 deficiency reduces sepsis-induced mortality associated with the attenuation of lung macrophages in mice
Source: Histochem Cell Biol. 2024 Aug 12;162(5):415–28. doi: 10.1007/s00418-024-02318-5 (PMC11393161; doi:10.1007/s00418-024-02318-5)

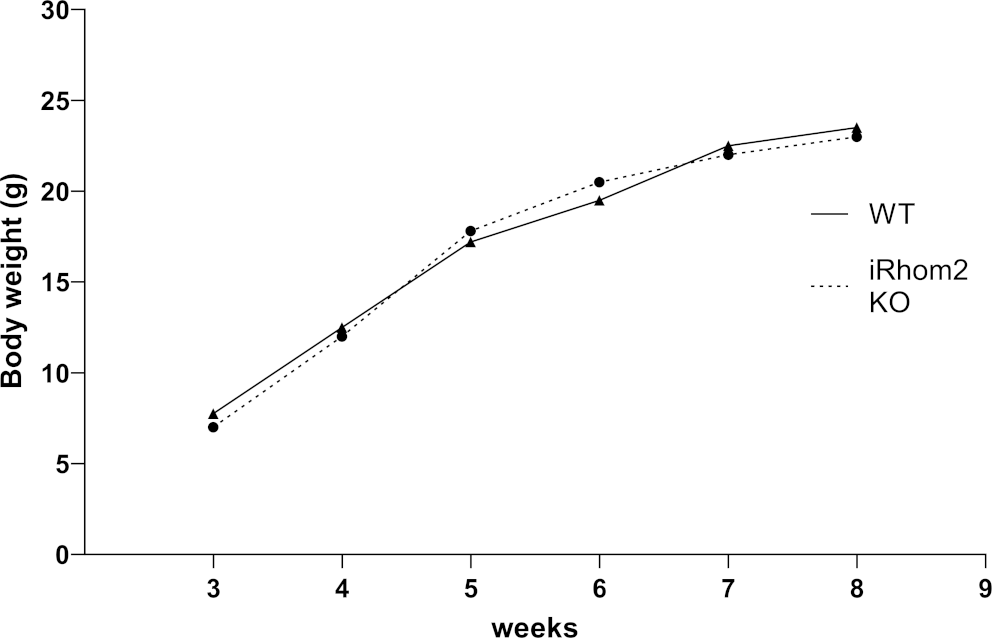

Supplement: Supplementary file 1 — Supplementary file1 (TIF 39 KB) [file 418_2024_2318_MOESM1_ESM.tif]
